# Local Optical Properties in CVD-Grown Monolayer WS_2_ Flakes

**DOI:** 10.1021/acs.jpcc.1c04287

**Published:** 2021-07-14

**Authors:** Michele Magnozzi, Theo Pflug, Marzia Ferrera, Simona Pace, Lorenzo Ramó, Markus Olbrich, Paolo Canepa, Hasret Ağircan, Alexander Horn, Stiven Forti, Ornella Cavalleri, Camilla Coletti, Francesco Bisio, Maurizio Canepa

**Affiliations:** † OptMatLab, Dipartimento di Fisica, Università di Genova, via Dodecaneso 33, 16146 Genova, Italy; ‡ Istituto Nazionale di Fisica Nucleare, Sezione di Genova, via Dodecaneso 33, 16146 Genova, Italy; § Laserinstitut Hochschule Mittweida, Technikumplatz 17, 09648 Mittweida, Germany; ∥ Technische Universität Chemnitz, Reichenhainer Str. 70, 09126 Chemnitz, Germany; ⊥ Center for Nanotechnology Innovation IIT@NEST, Piazza San Silvestro 12, 56127 Pisa, Italy; # Graphene Labs, Istituto Italiano di Tecnologia, Via Morego 30, 16163 Genova, Italy; ∇ Engineering Department, Istanbul Technical University, Maslak 34467, Istanbul, Turkey; ○ CNR-SPIN, C.so Perrone 24, 16152 Genova, Italy

## Abstract

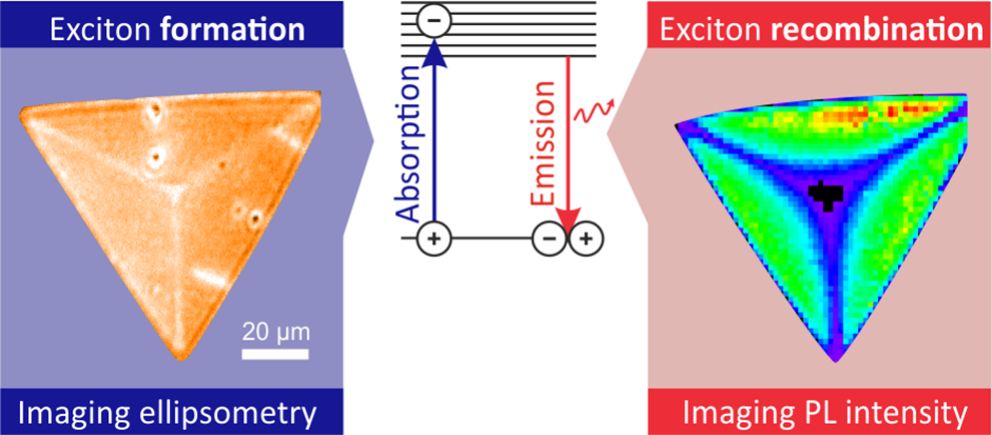

Excitons dominate the light absorption and re-emission spectra of monolayer transition-metal dichalcogenides (TMD). Microscopic investigations of the excitonic response in TMD almost invariably extract information from the radiative recombination step, which only constitutes one part of the picture. Here, by exploiting imaging spectroscopic ellipsometry (ISE), we investigate the spatial dependence of the dielectric function of chemical vapor deposition (CVD)-grown WS_2_ flakes with a microscopic lateral resolution, thus providing information about the spatially varying, exciton-induced light absorption in the monolayer WS_2_. Comparing the ISE results with imaging photoluminescence spectroscopy data, the presence of several correlated features was observed, along with the unexpected existence of a few uncorrelated characteristics. The latter demonstrates that the exciton-induced absorption and emission features are not always proportional at the microscopic scale. Microstructural modulations across the flakes, having a different influence on the absorption and re-emission of light, are deemed responsible for the effect.

## Introduction

Two-dimensional (2D) transition-metal dichalcogenides (TMD) are renowned for their extremely strong excitonic effects, which shape both their absorption and photoluminescence (PL) spectra.^1−4^ Many experiments revealed that the PL properties of single 2D TMD flakes are often spatially nonhomogeneous. In most cases, these variations were observed in TMD grown by chemical vapor deposition (CVD), which is currently one of the most effective methods to produce high-quality TMD for technological applications. Local variations in CVD-grown TMD can be intense and spectrally sharp, resulting in the enhancement, damping, and/or spectral shift of the PL features. Significant local variations have been reported between the edges and the center,^5−9^ along symmetry lines, resulting in inner segmentations,^10−15^ and in the case of nearby or coalesced flakes.^16^ These local modulations suggest intriguing possibilities to engineer diverse optical properties within single TMD flakes.^14,17,18^ On the other hand, much less effort was directed toward the study of the local modulation of the dielectric function of TMD, which is of fundamental importance to understand the light absorption properties and hence the creation of excitons in the material. Microscopic characterization of the primary absorption processes may be useful to understand and possibly exploit the physical mechanism underlying this phenomenon.

In this work, we investigated the excitonic properties of CVD-grown WS_2_ flakes tens of microns across by means of imaging spectral ellipsometry (ISE). Thanks to its high sensitivity provided by the detection of Ψ and Δ parameters, ISE allowed us to obtain very accurate imaging of the flake, revealing for the first time a richness of details on CVD-grown flakes not accessible through ordinary optical microscopy. By exploiting the analytical models previously designed and tested in the case of spatially averaged spectroscopic ellipsometry of WS_2_ flakes, we eventually obtained a mapping of the local variations in the dielectric function of WS_2_ with micrometric lateral resolution. This pushed naturally a comparison with PL mapping of comparable lateral resolution taken on the same flakes. The comparison showed many clear correspondences in the spatial patterns, highlighting the spatial excitonic structure of the flakes. This apparent correlation proved to be incomplete. On the one hand, regions exist that show clear excitonic features in the dielectric function but little to no PL signal. This fact is not completely surprising since nonradiative channels may be active in those regions. On the other hand, local increases in the PL intensity were observed in regions where absorption remains mostly constant. A critical analysis of the data suggests that structural defects in WS_2_, capable to differently modulate the light absorption or re-emission process, possess the characteristics needed to account for the effect.

## Methods

### Materials

WS_2_ single crystals were grown via liquid precursor CVD (LqP-CVD) using a process adapted from the previous work.^19^ First, Si/SiO_2_ used as substrate was cleaned via sonication it in acetone and isopropanol, respectively, for 5 min. Then, two aqueous solutions (solution A and solution B) containing ammonium metatungstate hydrate (AMT, Sigma-Aldrich, 463922), as tungsten precursor, and NaOH, as a growth promoter, were prepared by dissolving 0.15 g of AMT in 20 mL of deionized (DI) water and 0.1 g of NaOH in 40 mL of DI water, as a promoter, respectively. Then, solution A, solution B, and iodixanol, used as a density gradient (Sigma-Aldrich, Opti Prep, D1556), were mixed in a ratio of 1:2:0.3 and spin coated on a cleaned Si/SiO_2_ substrate. After spinning, WS_2_ was grown in a two-zone horizontal furnace (Lenton), where the substrate was heated at 800 °C in atmospheric pressure for 10 min under constant Ar/H_2_ flux, while sulfur was maintained at 120 °C. The as-grown WS_2_ single crystals were transferred on a 2 mm thick Suprasil substrate using a semidry transfer approach adapted from the previous work.^20^ In particular, the sample was covered by a polymeric membrane of poly(methyl methacrylate) (PMMA) and polypropylene carbonate (PPC), which were sequentially spun and baked at 90 °C for 2 min. Then, the sample was covered using a poly(dimethylsiloxane) (PDMS) frame and left floating in the water at 60 °C until the complete detachment of the membrane. Few drops of NaOH were also added to accelerate the detachment. After detachment, the floating membrane was deterministically transferred on the target using a transfer setup, as previously reported.^21^ Finally, the polymeric membrane was removed via a standard cleaning in acetone overnight.

### Experimental Methods

#### Imaging Spectroscopic Ellipsometry

Imaging spectroscopic ellipsometry was performed with a nanofilm_ep4 from Accurion GmbH using a 20× objective. The angle of incidence was set to 40° to maximize the signal on the detector. The 11 data points selected to obtain imaging spectral ellipsometry were acquired at 1.94, 1.97, 2.00, 2.03, 2.10, 2.32, 2.36, 2.41, 2.45, 2.50, and 2.85 eV. At each energy, two maps (Ψ and Δ) of 519 × 686 pixels were produced, corresponding to an area of 250 × 250 μm^2^. For the analysis of the flake discussed in the main text, the maps were cropped to 209 × 276 pixels, corresponding to an area of 100 × 100 μm^2^. From #1 to #5, the five regions of interest (ROIs) on that flake were composed of 1768, 1127, 383, 54, and 43 pixels, respectively. The resolution limit can be estimated as *d*_min_ = λ/*N*_a_, where λ is the wavelength in nm and *N*_a_ is the numeric aperture of the objective. We used *N*_a_ = 0.35; therefore, the resolution is 1.1 μm at 400 nm and 2.3 μm at 800 nm.

#### Imaging Photoluminescence and Raman Spectroscopies

Imaging photoluminescence and Raman spectra were acquired using a Jasco NRS-4100 confocal Raman spectrometer and a 100× objective. Photoluminescence spectra were obtained by irradiating the sample with a laser (λ = 532 nm; spot diameter = 1 μm; incident power = 25 μW) and setting the exposure time at 1 s. Spectral resolution: 0.3 meV. Raman spectra were acquired with the same laser parameters and spot size, exposure time = 100 s, spectral resolution = 0.017 meV.

#### Laterally Averaged Spectroscopic Ellipsometry

The spectroscopic ellipsometry data used to fit the optical model were acquired on a J.A. Woollam VASE at a 60° angle of incidence. The probe spot on the sample covered an area of 6.4 × 10^–2^ mm^2^ thanks to the use of microprobes. The spectral resolution was set to 1 nm, resulting in 224 data points from 1.9 to 2.9 eV. Depolarization ranged from 0.8% at 1.9 eV to 2.8% at 2.9 eV. The optical model was created on the WVASE 3.718 software (J.A. Woollam Co.).

#### Atomic Force Microscopy (AFM)

AFM experiments were carried out using a JPK NanoWizard IV microscope (Bruker). The morphology of the samples was obtained using gold-coated Si cantilevers (DNP-10, Bruker) with an elastic constant of 0.24 N/m. The AFM data were analyzed with JPKSPM data.

#### Note

All measurements are acquired at the nominal room temperature of 25 °C in air.

## Results and Discussion

**Figure 1:**
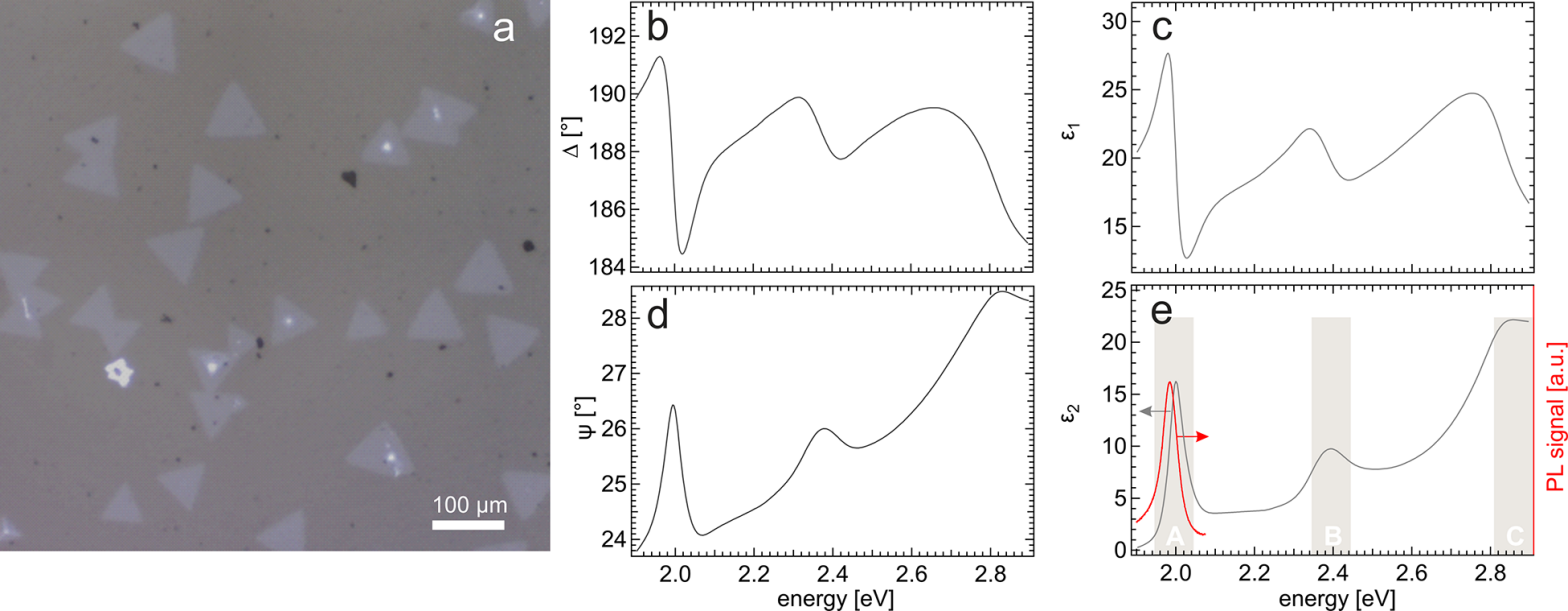
Laterally averaged excitonic properties of WS_2_ flakes. (a) Optical micrograph of the WS_2_ flakes on the silica substrate. (b, d) Δ and Ψ spectra obtained from laterally averaged ellipsometry measurements. (c, e) Real and imaginary parts of the laterally averaged dielectric function of WS_2_ obtained using the model described in ref (28). The laterally averaged photoluminescence spectrum is reported for comparison (red curve in panel e). The three bands in panel (e) indicate the spectral position of the A, B, and C excitons of WS_2_.

WS_2_ flakes were grown by CVD and transferred on a silica substrate, following a procedure detailed in the Methods section. The resulting flakes have a triangular shape, the typical size of tens of microns across, and are randomly distributed on the substrate (Figure 1a). We first performed a laterally averaged spectroscopic ellipsometry,^22−27^ acquiring Δ and Ψ spectra from an area of the sample containing ∼12 WS_2_ flakes (experimental details are reported in the Methods section). The laterally averaged Δ and Ψ spectra are reported in Figure 1b,d, respectively. Δ and Ψ are defined according to the equation
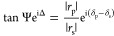
1where |*r*_p,s_|e^iδ_p_,δ_s_^ are the p, s-polarized complex Fresnel reflection coefficients of the system under investigation, Δ = δ_p_ – δ_s_ and Ψ = arctan |*r*_p_|/|*r*_s_|. The dielectric function ε = ε_1_ – iε_2_ can be extracted from Δ and Ψ by means of analysis. Using the same model and methodology as that described in ref (28), we therefore obtained the laterally averaged ε_1_ and ε_2_ of WS_2_ (Figure 1c,1e, respectively; see the Supporting Information I for details). The three excitonic features of WS_2_ are manifested as peaks in Ψ and the corresponding peak-and-trough shapes in Δ. They are located approximately at 2.0, 2.4, and 2.8 eV and conventionally labeled A, B, and C, respectively. The same kind of structures are present in ε_1_ and ε_2_. The results obtained from the laterally averaged ellipsometry data are instrumental to the interpretation of the ISE data on single WS_2_ flakes.

In Figure 1e, we plot a laterally averaged PL spectrum (red curve) on top of the ε_2_ curve to highlight the fact that the exciton formation and recombination determine the absorption and emission peaks, respectively. A spectral shift between the absorption and emission maxima (Stokes shift) occurs as a result of the structural disorder of WS_2_ and the n-type doping induced by the substrate.^29^

**Figure 2:**
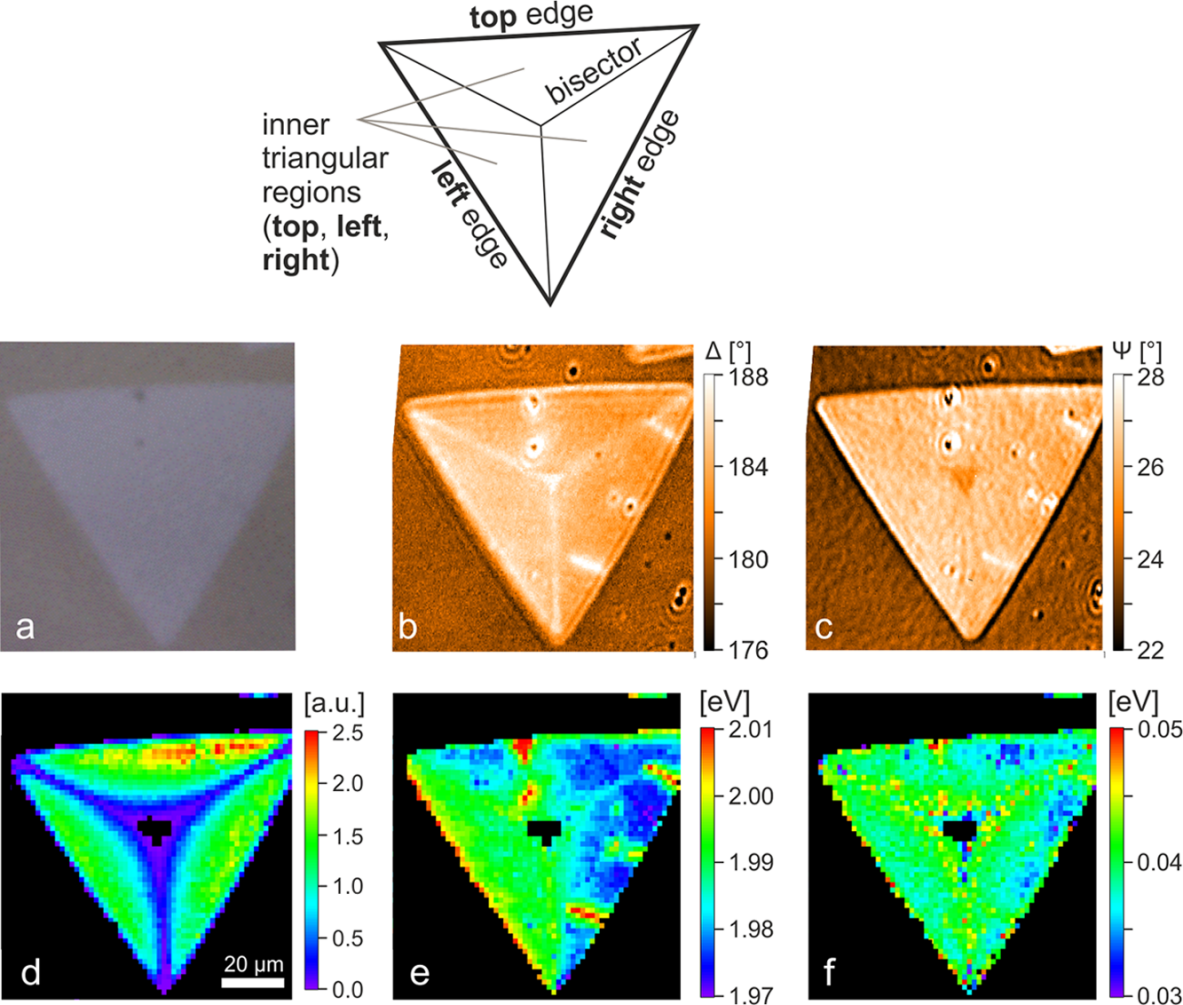
Microscopic characterization of the excitonic properties of one WS_2_ flake. Top: scheme of the flake indicating the landmarks used in the text. (a) Optical micrograph of the WS_2_ flake; (b, c) Δ and Ψ, respectively, resulting from imaging ellipsometry at 2.0 eV. (d–f) Intensity, spectral position, and FWHM, respectively, of the photoluminescence peak. Black pixels inside the flake in (d–f) are NaN.

To determine the local excitonic properties of WS_2_, we performed optical microscopy, imaging spectral ellipsometry (ISE), and imaging photoluminescence spectroscopy (IPL) on a single WS_2_ flake, allowing local variations to be resolved (the characterization of two more flakes is reported in the Supporting Information II). An image of the flake obtained with optical microscopy is reported in Figure 2a. To perform ISE, we used state-of-the-art instrumentation that allows us to reduce the lateral resolution of ellipsometry down to ∼1 to 2 μm (details in the Methods section). Indeed, ISE is a recent and powerful technical advancement of spectral ellipsometry, which has only recently begun to prove its value in the analysis of 2D systems^30−34^ and is generally well suited to probe local variations in the optical properties of materials.^35,36^ The ISE characterization consisted of 11 (Δ, Ψ) maps acquired between 1.94 and 2.85 eV, each map comprising ∼57,000 pixels. The ISE data at 2.0 eV (Figure 2b,c) were particularly relevant since that energy value was very close to the maximum excitonic absorption of WS_2_. The IPL maps, composed of 3600 pixels, were represented by plotting the intensity, spectral position, and full-width at half maximum (FWHM) resulting from a Lorentzian fitting of the PL peak (Figure 2d–f) (details on the IPL data fitting are reported in the Supporting Information III). The size of both ISE and IPL maps was approximately 100 × 100 μm^2^; additional details about the imaging measurements are reported in the Methods section.

While the flake appeared homogeneous by optical microscopy (Figure 2a), ISE and IPL revealed instead many local variations (Figure 2b–f). The landmarks used to identify specific regions or features of the flake are defined in Figure 2, top. The most noticeable local variations in Figure 2 were observed on the bisectors, in the center of the flake, and on two short segments close to its right edge. Moreover, the Δ (Figure 2b) and the spectral position of the PL peak (Figure 2e) suggested that the optical properties in the left inner triangular region were different with respect to those in the other two inner regions.

**Figure 3:**
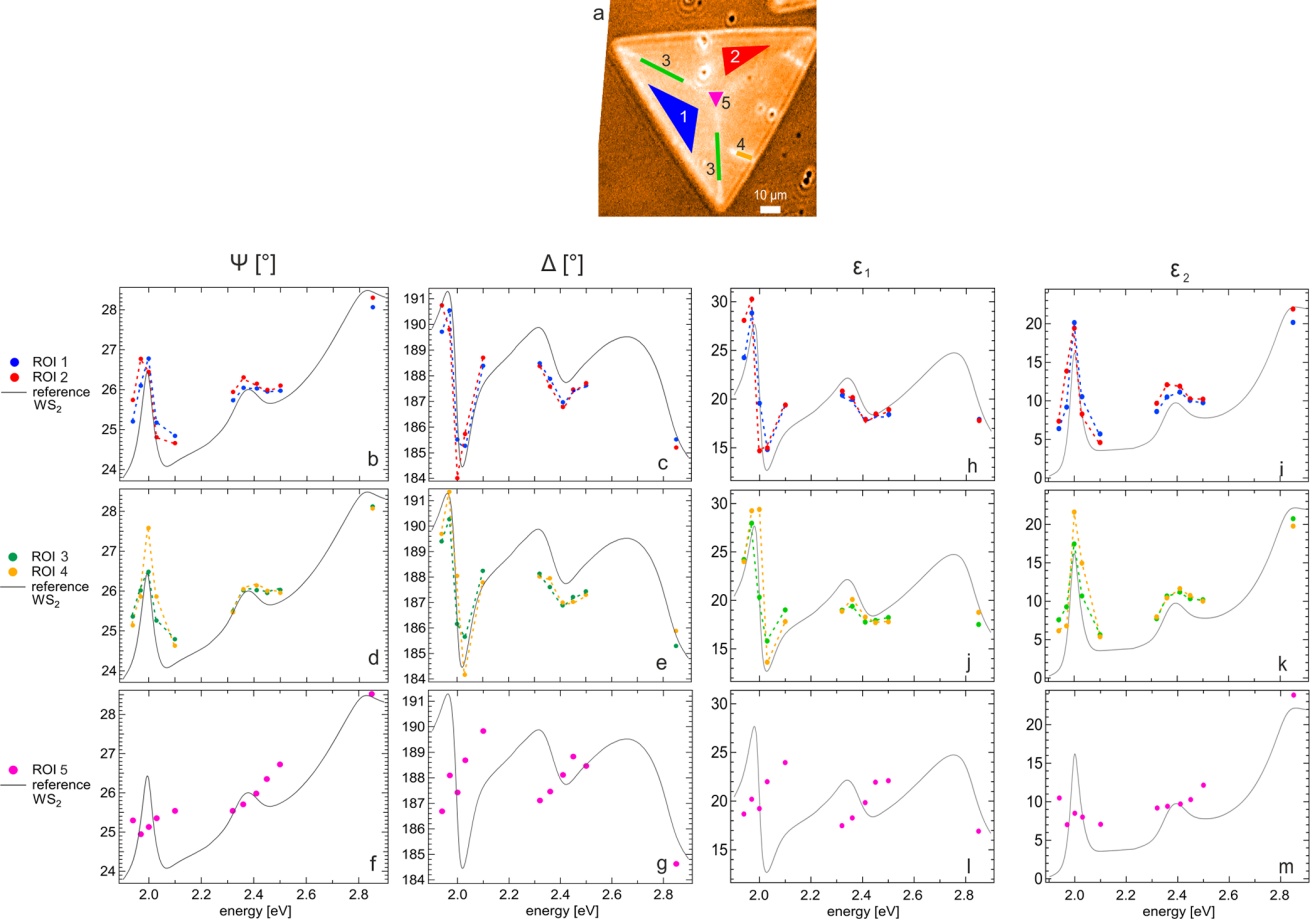
ROI-averaged ISE data and local dielectric function of the WS_2_ flake. (a) Δ map acquired at 2.0 eV, featuring the five ROIs considered in this work. (b, c, h, i) Ψ, Δ, ε_1_, and ε_2_, respectively, of ROIs 1 and 2. (d, e, j, k) Ψ, Δ, ε_1_, and ε_2_, respectively, of ROIs 3 and 4. (f, g, l, m) Ψ, Δ, ε_1_, and ε_2_, respectively, of ROI 5. Dotted lines between points are guides to the eye. The laterally averaged Ψ, Δ, ε_1_, and ε_2_ (gray curves) are reported for reference.

To obtain the local dielectric function from the ISE data, we identified five microscopic regions of interest (ROIs) on the ISE maps and calculated the average (Δ, Ψ) values in those regions. Once we know the substrate optical response and the WS_2_ thickness, then the only unknown parameters in the analysis are ε_1_ and ε_2_ of WS_2_; hence, they can be obtained from the ISE data using linear regression analysis without a dispersion model.^37^ The shape and location of the five ROIs were chosen according to the local variations observed in Figure 2b–f. The ROIs are sketched in Figure 3a. ROIs 1 and 2 are located within the left and top inner triangular regions, respectively; ROI 3 is composed of two regions encompassing the bisectors; ROI 4 is located around one of the two small segments adjacent to the right edge of the flake; and ROI 5 includes the center of the flake. The boundaries of the five ROIs were chosen to avoid image artifacts, like the diffraction patterns close to the flake edges. The ISE data averaged over each ROI are reported in Figure 3b–g. The resulting ε_1_ and ε_2_ averaged over each ROI are reported in Figure 3h–m.

The data from ROIs 1 to 4 reported in Figure 3 reveal the presence of the typical WS_2_ excitonic features; their spectral variations are made more evident by the dotted lines as a guide to the eye and by reporting the laterally averaged data (already reported in Figure 1b,1d) as a reference. Due to the correspondence between the ellipsometry spectra and the dielectric function of WS_2_, the trends of local variations observed in the two data sets are fully consistent. The A exciton (1.9–2.1 eV) underwent sizable intensity and spectral variations depending on the considered ROI: in ROI 2, it was red shifted with respect to ROI 1 (Figure 3b,c,h,i); in ROI 3, its intensity was slightly smaller, with no spectral shift; however, in ROI 4, its intensity was larger and a blue-shift occurred (Figure 3d,e,j,k). The other excitonic features around 2.4 and 2.8 eV showed no clear effect, thus confirming the results recently reported in ref (29). Finally, ROI 5 showed no excitonic features (Figure 3f,g,l,m). The spectral resolution that we report is given by the instrument characteristics. To better appreciate the small, yet detectable spectral shift among different ROIs, we must therefore look at all data points, not only the maximum value, and consider both the ellipsometric and the dielectric function curves. By determining the ε_1_ and ε_2_, we obtained two results: first, we determined the local dielectric function of CVD-grown WS_2_ flakes (so far, only the laterally averaged dielectric function has been reported on that system); second, we proved that the local variations in the Δ, Ψ maps are a good estimator of the variations in the dielectric function; therefore, we can consider those two data sets as interchangeable for our purposes (i.e., they provide the same kind of information about the excitonic features).

Having identified the local variations in the absorption-related data (ISE, dielectric function), we now compare them to the emission-related data (IPL). A high correlation exists between the two data sets, as both ISE and IPL techniques revealed: (i) the damping or suppression of the A excitonic features in the center of the flake and along the bisector; (ii) slightly different properties of the left inner triangle with respect to the rest of the flake; (iii) sharp local variations in the two segments along the right side of the flake (the latter features are caused by small cracks in the flake, see Figure SI5 in the Supporting Information). A strong correlation was found between the spectral shifts in the energy of the excitonic absorption, detected by ISE, and corresponding shifts in the energy of the PL peak (Figure 2e). A qualitative similarity was also observed between the ISE maps and the IPL intensity along the bisectors, as the intensity of both the IPL peak and the excitonic absorption peak was damped with respect to the rest of the flake, and at the center of the flake, where the IPL signal was absent and ISE data show no excitonic feature.

Given the remarkable similarities described above in the ISE and IPL data, it would be tempting to conclude that the local dielectric function is a robust predictor of the photoluminescence signal and vice versa. Our data, however, show that this was not always the case: indeed, a few significant differences between the ISE and IPL maps point toward a richer and more complex picture of the optical properties of WS_2_. Notably, the spatial extension of the local variations at the center and along the bisectors in the ISE maps was generally smaller than that in the IPL maps. Moreover, a few local features in the IPL intensity map were not present in the ISE data and vice versa. For example, there is a sizable maximum of the PL intensity in the region close to the top edge, while in the same region, no variations are detected by ISE; and the regions featuring the maximum absorption (ROI 4) show no variations in the PL intensity. This lack of correlation demonstrates that it is possible to spatially decouple the excitonic absorption and emission maxima on a microscopic scale within individual TMD flakes, a finding that has implications both for the fundamental understanding of the excitonic behavior and the engineering of spatially varying excitonic properties in the monolayer TMD.

A few considerations can be made about the physical origin of the observed local variations in the WS_2_ flakes; in doing so, we first rule out a few unlikely explanations, then present our most likely hypotheses. Notably, all of the regions where local variations occurred have well-defined shapes in both the ISE and IPL data and are extended over several μm^2^ (therefore, much larger than the spatial resolution of both ISE and IPL); moreover, the optical response of the sample did not change even after repeated data acquisitions. These characteristics allowed us to rule out random dielectric disorder^38^ and laser-induced effects.^12,39−41^ Spurious effects due to the growth promoters can be ruled out as well since they were all dissolved during the transfer. Moreover, the WS_2_ height measured by AFM (Supporting Information IV) was remarkably uniform across the flake, meaning that none of the observed variations can be ascribed to the topological deviations from the monolayer (this was further confirmed by Raman spectra, see the Supporting Information V). We also note that the same kind of trends observed on the flake in Figure 2 were replicated on other WS_2_ flakes from the same sample (Supporting Information II).

The local variations observed in the ISE and IPL data in the center of the flake and along the bisectors can be explained by considering that in those regions, structural and charged defects such as sulfur vacancies^10,42^ are known to occur, which may also define grain boundaries along the directions defined by the bisectors.^11,16,43^ In particular, the lack of excitonic features in the central region of the flake (ROI 5) is attributed to the high density of defects, which naturally occur at the nucleation centers.^10,44^ The qualitative difference of the central region is also demonstrated by micro-Raman measurements (Supporting Information V). The IPL intensity enhancement observed along the top edge of the flake is tentatively attributed to oxygen chemisorption and physisorption;^7^ as stated before, it is noteworthy that this kind of local variation is not detected by ISE, nor it is correlated to the spectral position of the IPL peak. Indeed, the overall qualitative diversity of the IPL intensity map may be explained by considering that defects in the WS_2_ structure^45^ are known to introduce efficient nonradiative channels,^42^ which may ultimately cause the light emission from WS_2_ (probed by IPL) to be quite different from the light absorption (probed by ISE). Ultimately, it can be argued that the differences observed with the two techniques are intimately linked to the different transitions they probe, which in turn determine their different sensitivities toward the presence of defects or adsorbates. In principle, this feature could be instrumental to tailor almost separately the absorption and emission intensity at a microscopic scale, thus engineering diverse optical properties within individual TMD flakes.

## Conclusions

In summary, we have applied imaging spectroscopic ellipsometry (ISE) measurements to probe the local dielectric properties within individual CVD-grown monolayer WS_2_ flakes. ISE, thanks to the phase sensitivity (so-called Δ parameter), revealed a richness of details at the microscopic scale, which is inaccessible to ordinary microscopy. ISE therefore shows itself as a powerful diagnostic tool that can be beneficial toward the engineering and exploitation of diverse optical properties in monolayer TMD flakes. The high spatial resolution of ISE made the comparison between spatially resolved exciton-related absorption with IPL measurements obtained with comparable lateral resolution feasible. The energy of the main exciton (so-called A exciton) in the local dielectric function correlated very well with the energy of the PL peak. Overall, ISE and IPL patterns show evident correlations. However, the spatial variations in the intensity of the A exciton are not always matched by equivalent variations in the corresponding PL intensity signal and vice versa. Local structural defect patterns, induced by the WS_2_ flake growth dynamics, induce different degrees of modulations of the light absorption and light re-emission characteristics and therefore represent the most suited physical mechanism accounting for the observations.

## Supporting Information Available

I: Modeling the laterally averaged SE data; II: microscopical characterization of three WS_2_ flakes; III: IPL data fitting; IV: AFM data; and V: Raman spectra (PDF)
