# Supplementary material for: Local Optical Properties in CVD-Grown Monolayer WS2 Flakes
Source: J Phys Chem C Nanomater Interfaces. 2021 Jul 14;125(29):16059–65. doi: 10.1021/acs.jpcc.1c04287 (PMC8411805; doi:10.1021/acs.jpcc.1c04287)
Supplement: Supplementary file 1 — jp1c04287_si_001.pdf [file jp1c04287_si_001.pdf]

Local Optical Properties in CVD-Grown Monolayer WS_2 Flakes

Michele Magnozzi,[1,2] Theo Pflug,[3,4] Marzia Ferrera,[2] Simona Pace,[5,6] Lorenzo Ramo',[2] Markus Olbrich,[3] Paolo Canepa,[2] Hasret Ağircan,[7] Alexander Horn,[3] Stiven Forti,[5,6] Ornella Cavalleri,[2] Camilla Coletti,[5,6] Francesco Bisio[8], and Maurizio Canepa[2]

[1] *OptMatLab, Dipartimento di Fisica, Università di Genova, via Dodecaneso 33, 16146 Genova, Italy*

[2] *Istituto Nazionale di Fisica Nucleare, Sezione di Genova, via Dodecaneso 33, 16146 Genova, Italy*

[3] *Laserinstitut Hochschule Mittweida, Technikumplatz 17, 09648 Mittweida, Germany*

[4] *Technische Universität Chemnitz, Reichenhainer Str. 70, 09126 Chemnitz, Germany*

[5] *Center for Nanotechnology Innovation IIT@NEST, Piazza San Silvestro 12, 56127 Pisa, Italy*

[6] *Graphene Labs, Istituto Italiano di Tecnologia, Via Morego 30, 16163 Genova, Italy*

[7] *Engineering Department, Istanbul Technical University, Maslak, 34467, Istanbul, Turkey*

[8] *CNR-SPIN, C.so Perrone 24, 16152 Genova, Italy*

Supporting Information I

Modelling the laterally-averaged SE data

In order to model the ellipsometry data, we adopted the following strategy. On the same sample considered in the main text, we performed an SE measurement with a J.A. Woollam VASE equipped with focusing probe; then, we adapted the model presented in Ref. [1] to reproduce the experimental data from VASE. Briefly, we used a patented PSEMI dispersion formula (US patent 5,796,983, Aug. 18, 1998, Herzinger et al.) to describe the WS_2 optical response, and used a linear Effective Medium Approximation to properly take into account the surface coverage of WS_2. In this way, we obtained a model that can satisfactorily describe the "average" optical properties of the WS_2 flake described in the main text. In the following, we review in detail each step.

The structure of the model (i.e. number of layers and oscillators) is the same of Ref. [1]. The substrate was measured with SE before the WS_2 deposition, and its ellipsometric response was modelled independently with a Cauchy dispersion formula; the amplitude and center position of each oscillator in the WS_2 layer was fitted to the VASE data. We fed into the model the actual surface coverage of WS_2 in the area probed by VASE, that is, 30% (this value was obtained from the analysis of optical microscopy images), and the WS_2 thickness (0.8 nm, obtained from AFM data in Supporting Information IV). Therefore, the model was effectively adapted to describe the specific sample under investigation.

The accuracy of the model was good (MSE=5.4); the experimental and calculated data are compared in Fig. SI1. Indeed, the model reproduces very well the features corresponding to the A and B excitons, while at the higher energies, the accuracy seems to decrease. However, it must be noted that due to depolarization and low intensity on the detector, the experimental datapoints at higher energies have a relatively large uncertainty (at least ±1 in Ψ and ±2.5 in Δ); for this reason, the relative weight of those datapoints within the fitting calculations is smaller, resulting in a higher discrepancy between generated and experimental data.

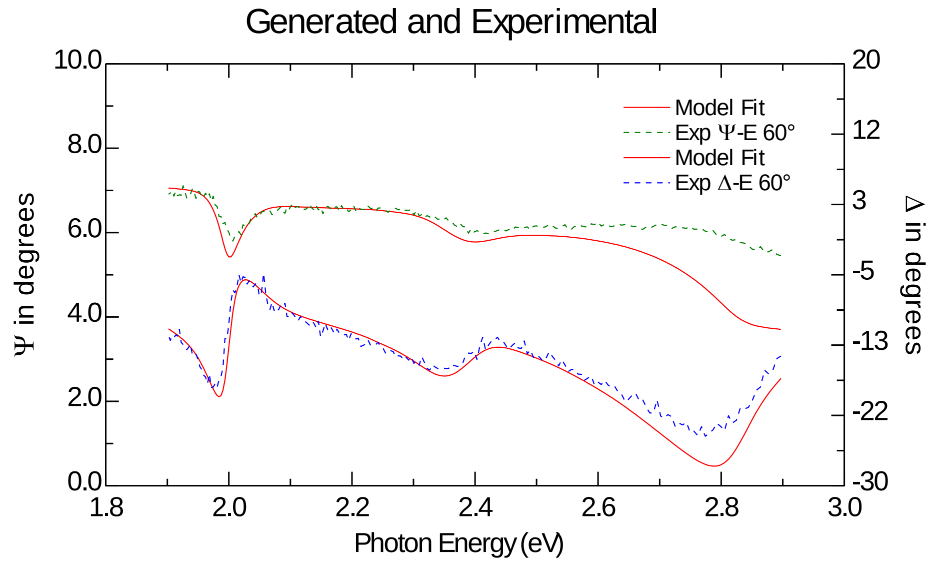

Fig. SI1. Dotted curves: Experimental Ψ and Δ measured with VASE; Continuous curves: Ψ and Δ calculated from the model adapted from Ref. [1].

The validated model allows to generate ellipsometry data that can be compared with the ISE data reported in the main text. There, we calculated SE data by considering a 100% surface coverage of WS_2 and 40° angle of incidence, in order to match the conditions in which the ISE data were acquired. From the model, we also calculate the complex dielectric function of monolayer WS_2 in the case of 100% surface coverage. In both cases, the analysis on the laterally-averaged SE data provides an “average” reference for the ISE data and local dielectric function.

[1] Magnozzi, M. *et al*. Optical dielectric function of two-dimensional WS_2 on epitaxial graphene. *2D Materials*, 7, 025024, 2020.

Supporting Information II

Microscopical characterization of three WS_2 flakes

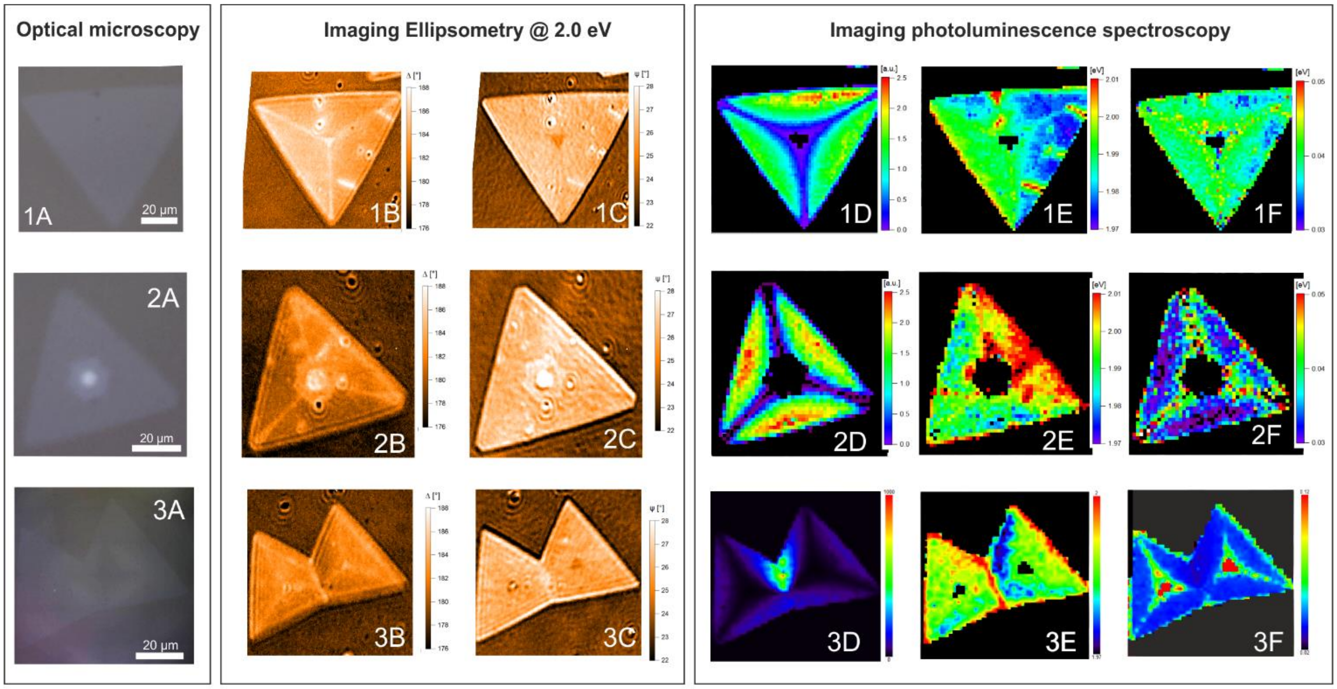

Fig. SI2: overview of three WS_2 flakes observed with optical microscopy (column A), imaging ellipsometry at 2.0 eV (col. B: Δ; col. C: Ψ), and photoluminescence spectroscopy (col. D: peak intensity; col. E: spectral position; col. F: FHWM).

Supporting Information III

IPL data fitting

The PL spectra of monolayer WS_2 on SiO_2 typically exhibit a major peak and much less intense, broader, and redshifted one; they are determined by the neutral excitons and charged excitons (trions), respectively. The two peaks are represented in Fig. SI3 as two Lorentzian functions fitted to one IPL spectrum. In the IPL data of this work, the trion peak was often so small that it became undistinguishable from the background; therefore, only the main peak (neutral exciton) is discussed in the main text.

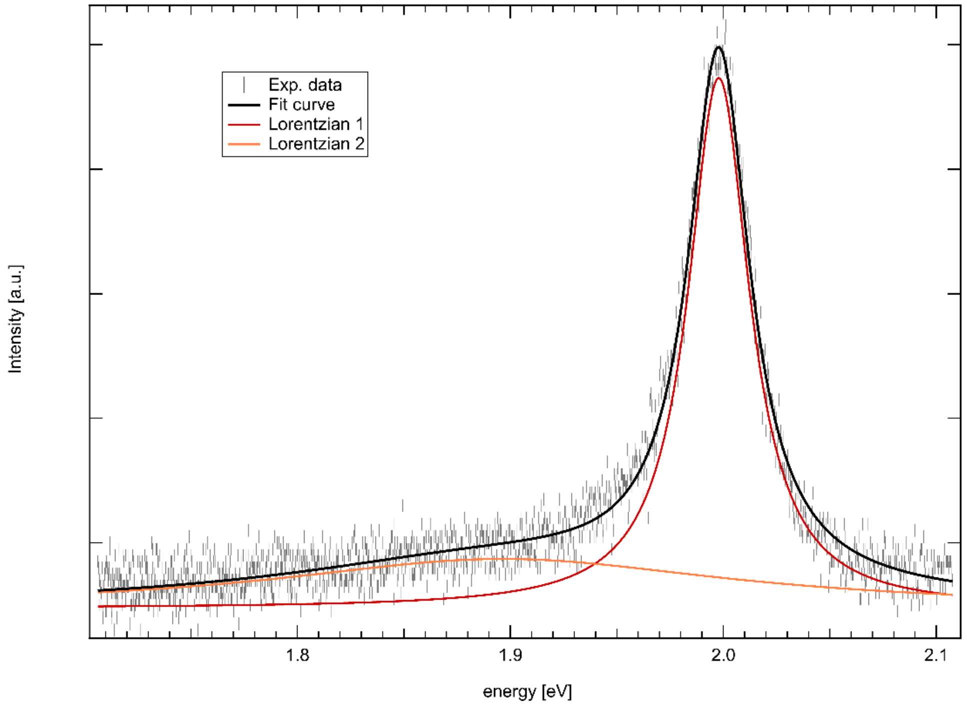

Fig. SI3. In this IPL spectrum, the trion feature is sufficiently intense to be distinguished from the background.

Supporting Information IV

AFM Data

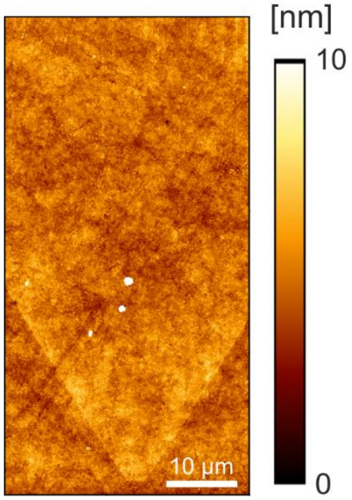

Fig. SI4. AFM height signal from the flake discussed in the main text. A remarkably uniform height was observed on the whole flake, with no multilayer terraces.

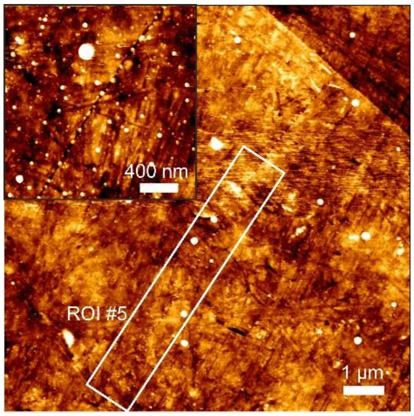

Fig. SI5. ROI 4 (white rectangle) observed with AFM. The whole ROI is crossed by a crack in WS_2 which extends up to the edge of the flake. The inset shows a zoom on the crack.

Supporting Information V

Raman spectra

Representative Raman spectra confirmed that the flake is composed of a monolayer WS_2. The Raman spectrum acquired within the top inner triangular region (red dot in Fig. SI6) describes the typical fingerprint of a monolayer WS_2, as indicated by the Raman mode $(2LA(M)+E^1{}_{2g})/A_{1g}$ intensity ratio greater than 5. [2,3] The same spectral pattern is measured on the bisector. In the center of the flake, however, the features depart from those of typical monolayer WS_2 $((2LA(M)+E^1{}_{2g})/A_{1g} \sim 2)$, due to the fact that this region corresponds to the center of nucleation, where structural defects are formed during the growth of the flake. [4]

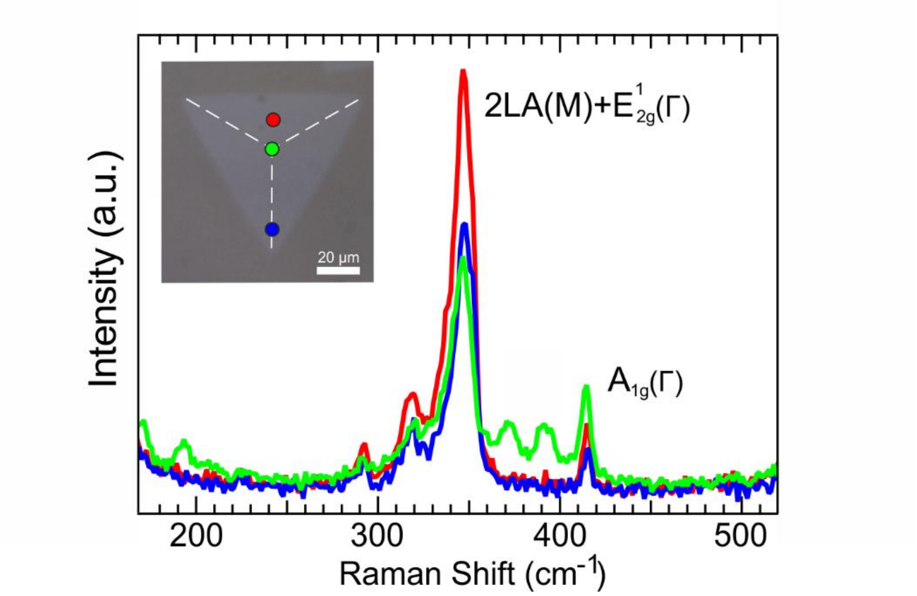

Fig. SI6. Raman spectra acquired on the flake discussed in the main text.

[2] Berkdemir, A. et al. Identification of individual and few layers of WS_2 using Raman Spectroscopy. *Scientific Reports* 3**,** 1755 , 2013.
[3] Pace, S. et al. Thermal stability and photo-activated degradation of monolayer WS_2 in BEOL conditions. *Journal of Physics: Materials*, 4, 024002, 2021.
[4] Cong, C. et al. Optical properties of large-area single-crystalline 2D semiconductor WS_2 monolayer from chemical vapor deposition. *Advanced Optical Materials* 2, 131, 2014.
